# Genetic structure and ecological niche segregation of Indian gray mongoose (*Urva edwardsii*) in Iran

**DOI:** 10.1002/ece3.8168

**Published:** 2021-09-29

**Authors:** Razie Oboudi, Mansoureh Malekian, Rasoul Khosravi, Davoud Fadakar, Mohammad Ali Adibi

**Affiliations:** ^1^ Department of Natural Resources Isfahan University of Technology Isfahan Iran; ^2^ Department of Natural Resources School of Agriculture Shiraz University Shiraz Iran; ^3^ Department of the Environment Semnan Provincial Office Semnan Iran

**Keywords:** climate change, ecological niche modeling, genetic structure, Indian gray mongoose, mitochondrial DNA, niche divergence

## Abstract

Combining genetic data with ecological niche models is an effective approach for exploring climatic and nonclimatic environmental variables affecting spatial patterns of intraspecific genetic variation. Here, we adopted this combined approach to evaluate genetic structure and ecological niche of the Indian gray mongoose (*Urva edwardsii*) in Iran, as the most western part of the species range. Using mtDNA, we confirmed the presence of two highly differentiated clades. Then, we incorporated ensemble of small models (ESMs) using climatic and nonclimatic variables with genetic data to assess whether genetic differentiation among clades was coupled with their ecological niche. Climate niche divergence was also examined based on a principal component analysis on climatic factors only. The relative habitat suitability values predicted by the ESMs for both clades revealed their niche separation. Between‐clade climate only niche comparison revealed that climate space occupied by clades is similar to some extent, but the niches that they utilize differ between the distribution ranges of clades. We found that in the absence of evidence for recent genetic exchanges, distribution models suggest the species occurs in different niches and that there are apparent areas of disconnection across the species range. The estimated divergence time between the two Iranian clades (4.9 Mya) coincides with the uplifting of the Zagros Mountains during the Early Pliocene. The Zagros mountain‐building event seems to have prevented the distribution of *U. edwardsii* populations between the western and eastern parts of the mountains as a result of vicariance events. Our findings indicated that the two *U. edwardsii* genetic clades in Iran can be considered as two conservation units and can be utilized to develop habitat‐specific and climate change‐integrated management strategies.

## Graphical abstract

Combining genetic data with ecological niche modeling is an effective approach for defining conservation units and exploring environmental variables that affect spatial patterns of genetic variation. We found two distinct genetic clades within *H. edwardsii* significantly correlated with different climate features. Niche‐based distribution models revealed that the two molecular clades occupy distinct environmental spaces, which are not similar, and equivalent.

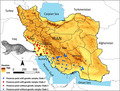

## 1 INTRODUCTION

A number of biological and ecological processes are known to shape patterns of genetic structure in continuous or discrete populations of species such as geographic, ecological, or reproductive barriers (e.g., Barton, 2008; Bradburd et al., 2013). Landscape models have been developed to examine the roles of landscape, that is, isolation by resistance (McRae & Beier, 2007), and environmental niche, that is, isolation by environment (Wang & Summers, 2010) on gene flow. Despite the importance of environmental niche dissimilarity as a motivator of genetic differentiations, its dynamism and strength are poorly known.

Spatially explicit environmental data in the framework of ecological niche models (ENMs) have been widely used to generate geographic predictions of a species’ distribution (Guisan & Thuiller, 2005). However, in ENMs, a genetic uniformity throughout a species’ range is assumed and the potential for local adaptation to specific environmental conditions is ignored (Gotelli & Stanton‐Geddes, 2015; Grady et al., 2011, 2013). Research has been shown that incorporating information on genetic variability and local adaptation improves model performance (e.g., Ikeda et al., 2017; Marcer et al., 2016) and helps to identify processes that have shaped population structure of species in the past and present (e.g., Alvarado‐Serrano & Knowles, 2014; Chang et al., 2012; Knowles et al., 2007; Marcer et al., 2016; May et al., 2011).

Ecological niche differences among species or populations can be analyzed to evaluate the possible ecological and evolutionary forces that shape geographic distributions, habitat preferences, and genetic structures (e.g., Raxworthy et al., 2007). A combination of ecological niche models and genetic analysis has potentially broad applications for defining conservation units, including evolutionary significant units (ESUs; e.g., Crandall et al., 2000), management units (MUs), and distinct population segments (DPSs; The Endangered Species Act, 1978) for a broad range of taxa.

Here, we employed an integrative approach to explore genetic and ecological differentiations within the Indian gray mongoose (*Urva edwardsii*, Geoffroy Saint‐Hilaire 1818) distributed along the southern part of Iran. Southern Iran consists of the southern mountain ranges of Zagros, Central Iranian Range, Khuzestan Plain, and the northern coasts of Persian Gulf.

The southern Zagros mountainous ridge has been regarded as one of the most known natural barriers between central Iran and the Mesopotamian plain to the west, responsible for the vicariance event in the region for different taxa (e.g., Ghaedi et al., 2020; Nazarizadeh et al., 2016). The presence of unsuitable environmental conditions or geographic distance may limit gene flow among populations, resulting in different genetic clades; thus, the present distributional patterns of species may be due to vicariant events (Nazarizadeh et al., 2016; Nilson et al., 2003). Climate also act as the primary driver of adaptive divergence and shaping genetic evolution, by limiting individuals’ establishment and dispersal (Pearson & Dawson, 2003). However, there is no information as to whether the Zagros Mountains have acted as a barrier for small carnivores such as mongoose species.

Smaller carnivores such as mongooses lack the attraction and attention that managers and conservationists seek in mega‐carnivores as suitable flagship species and conservation tools. Thus scientific information on small carnivores such as the Indian gray mongoose is still scarce in many countries including Iran. Small carnivores, however, show stronger specialization and resource selectivity compared to large carnivores (Kalle et al., 2012), and then, they may serve as useful indicator species in the preservation of keystone habitats. Knowledge on spatial scale and landscape heterogeneity is integral to the understanding of species habitat associations. Several factors including trapping for meat or fur (used in shaving and paint brushes), and good luck charms threaten the viability of mongooses (Mallick, 2009). In addition, limited data are available on the taxonomic status, habitat requirements, ecological niche differentiations, and genetic distances among the populations of *U. edwardsii* across its distribution range, which hinders the effective management of the species. Therefore, the current study aimed to (i) provide insights into intraspecific genetic diversity and niche differentiations among *U. edwardsii* populations in Iran and (ii) assess whether the Zagros Mountain may have acted as a physical barrier between the Indian gray mongoose populations.

## 2 METHODS

### 2.1 Study area

**FIGURE 1:**
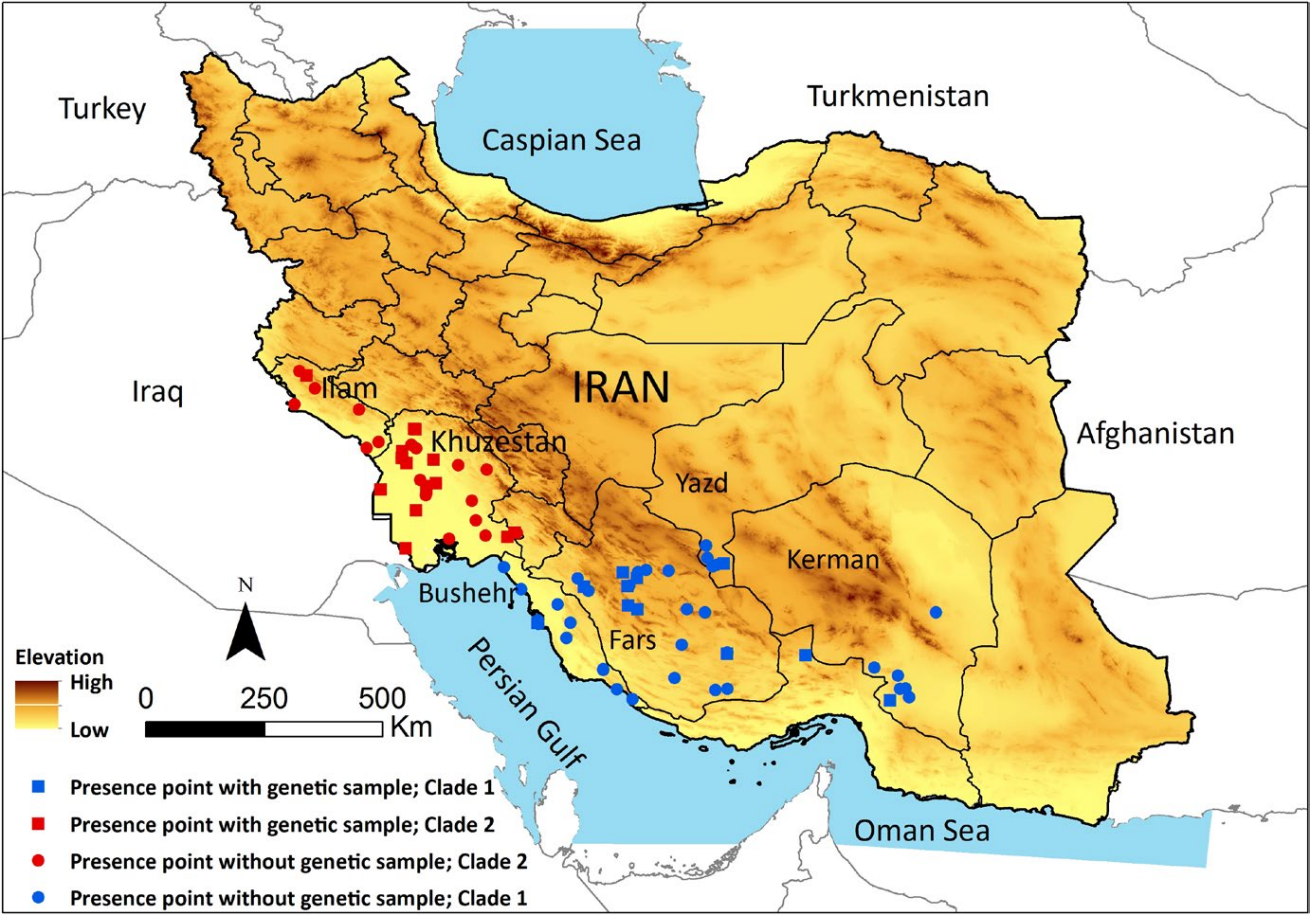
Genetic samples and species presence localities of Indian gray mongoose in south–southeast (Clade 1, blue) and southwest of Iran (Clade 2, red)

The study area covers the distribution range of Indian gray mongoose in Southern Iran (Ziaie, 2008) (Figure 1). The species inhabits a variety of habitats including meadows, sparse woodlands, shrublands, croplands, habitats near to reedy wetland, and palm trees near villages (Karami et al., 2016). This region, which is mainly located in zoogeographic realm of Saharo‐Arabian (Holt et al., 2013), is affected by subequatorial climate condition with cold and rainy winter and warm and dry summer (Heshmati, 2012). The annual precipitation ranges from 50 to 650 mm with an increasing gradient from east to west. Mean annual temperature ranges from 2 to 28°C, where January and February are the coldest, and July and August are the warmest months.

*Urva edwardsii* has recently been introduced into some parts of Central Iran such as Isfahan Province (Yusefi et al., 2019). While *U. edwardsii* is found from Central to Southern Iran, the small Indian mongoose (*Urva auropunctata,* Hodgson 1836) only reported from Sistan and Baluchestan Province (Sistan region), Hormozgan Province, and few localities in Khuzestan Province (Yusefi et al., 2019 and references therein). The species was also introduced to many islands and mainland to acts as a biological control of rats and snakes in plantations (Barun et al., 2013; Simberloff et al., 2000; Thulin et al., 2006), and it is considered as one of the worst invasive alien species (Lowe et al., 2000).

#### 2.1.1 Sampling, DNA extraction, and sequencing

To assess historical relationships among *U. edwardsii* populations, 24 specimens were collected across the species range in Iran (Figure 1). In addition, we collected three samples of *U. auropunctata* from southwest of Iran. All samples were obtained from road kills or natural cause deaths between 2016 and 2018 and stored in 98% ethanol. Also, 17 control region sequences of *Urva javanica, U. edwardsii,* and *U. auropunctata* were obtained from GenBank. The locality information, ID, and accession numbers are summarized in Table 1.

**TABLE 1:** List of mongoose samples used in the current study

No.	Species	Sample ID	Country	Location	Reference	Accession no.
1	*Urva edwardsii*	H1_FARS1	Iran	Firuzabad	This study	MZ043130
2	*U. edwardsii*	H1_FARS2	Iran	Beyza	This study	MZ043131
3	*U. edwardsii*	H1_FARS3	Iran	Beyza	This study	MZ043132
4	*U. edwardsii*	H1_FARS4	Iran	Beyza	This study	MZ043133
5	*U. edwardsii*	H1_FARS5	Iran	Lapouyee	This study	MZ043134
6	*U. edwardsii*	H1_FARS6	Iran	Shiraz	This study	MZ043135
7	*U. edwardsii*	H1_KRMN1	Iran	Manoojan	This study	MZ043136
8	*U. edwardsii*	H1_KRMN2	Iran	Hajiabad	This study	MZ043137
9	*U. edwardsii*	H1_YAZD1	Iran	Harat	This study	MZ043138
10	*U. edwardsii*	H1_BSHR1	Iran	Bushehr	This study	MZ043139
11	*U. edwardsii*	H2_FARS7	Iran	Shiraz	This study	MZ043140
12	*U. edwardsii*	H3_FARS8	Iran	Zarindasht	This study	MZ043141
13	*U. edwardsii*	H4_KZTN1	Iran	Dasht‐eazadegan	This study	MZ043142
14	*U. edwardsii*	H4_KZTN2	Iran	Karkheh protected area	This study	MZ043143
15	*U. edwardsii*	H4_KZTN3	Iran	Dezful	This study	MZ043144
16	*U. edwardsii*	H5_KZTN4	Iran	Dezful	This study	MZ043145
17	*U. edwardsii*	H5_KZTN5	Iran	Shush	This study	MZ043146
18	*U. edwardsii*	H5_KZTN6	Iran	Shush	This study	MZ043147
19	*U. edwardsii*	H6_KZTN7	Iran	Shushtar	This study	MZ043148
20	*U. edwardsii*	H6_KZTN8	Iran	Behbahan	This study	MZ043149
21	*U. edwardsii*	H6_KZTN9	Iran	Behbahan	This study	MZ043150
22	*U. edwardsii*	H7_KZTN10	Iran	Ahvaz	This study	MZ043152
23	*U. edwardsii*	H8_KZTN11	Iran	Molasani	This study	MZ043153
24	*U. edwardsii*	H9_Ilam1	Iran	Abdanan	This study	MZ043151
25	*Urva auropunctata*	HA1_KZTN12	Iran	Abadan	This study	MZ043129
26	*U. auropunctata*	HA1_KZTN13	Iran	Ahvaz	This study	MZ043128
27	*U. auropunctata*	HA1_FARS9	Iran	Shiraz	This study	MZ043127
28	*U. edwardsii*	UeFJ687485	Italy	Italy	Gaubert and Zenatello (2009)	FJ687485
29	*U. edwardsii*	UeTC144	Bahrain	Sharjah Desert Park	Veron and Jennings (2017)	KY346596
30	*U. edwardsii*	UeTC145	Bahrain	Sharjah Desert Park	Veron and Jennings (2017)	KY346597
31	*U. edwardsii*	UeTC146	U.A.E	Sharjah Desert Park	Veron and Jennings (2017)	KY346598
32	*U. edwardsii*	UeTC148	U.A.E	Sharjah Desert Park	Veron and Jennings (2017)	KY346599
33	*U. edwardsii*	UeTC294	Bangladesh	Payradanga	Veron and Jennings (2017)	KY346600
34	*U. edwardsii*	UeTC295	Bangladesh	Payradanga	Veron and Jennings (2017)	KY346601
35	*U. edwardsii*	UeTC296	Bangladesh	Payradanga	Veron and Jennings (2017)	KY346602
36	*Urva javanica*	UjB17400	China	Canton, China	Veron and Jennings (2017)	KY346603
37	*U. javanica*	UjTC472	Vietnam	Cuc Phuong National Park	Veron and Jennings (2017)	KY346604
38	*U. javanica*	T170_Thai	Thailand	Thailand	Gaubert et al. (2011)	GU183515
39	*U. auropunctata*	UaFJ411052	Jamaica	Jamaica	Bennett et al. (2009)	FJ411052
40	*U. auropunctata*	UaTC142	Guyana	Georgetown	Veron and Jennings (2017)	KY346591
41	*U. auropunctata*	UaTC220	Myanmar	Myanmar Zoo	Veron and Jennings (2017)	KY346592
42	*U. auropunctata*	UaTC221	Myanmar	Myanmar Zoo	Veron and Jennings (2017)	KY346593
43	*U. auropunctata*	UaTC297	Bangladesh	Dhaka	Veron and Jennings (2017)	KY346594
44	*U. auropunctata*	UaTC298	Pakistan	Pakistan	Veron and Jennings (2017)	KY346595
45	*Herpestes brachyurus*	–	–	–	Salleh et al. (2017)	KY117547

Whole genomic DNA was extracted from the tissue samples using WizPrep^TM^ gDNA Mini Kit (Cell/Tissue), following the manufacturers’ instructions. Polymerase chain reactions (PCRs) were performed for the amplification of a 691 base pairs (bp) fragment of the mitochondrial control region using HERP–DL‐93‐L (5' CAACTCCACCCCACAACTCT 3'; Gaubert & Zenatello, 2009) and HERP–DL656H (5' TGTGTGATCATGGGCTGATT 3'; Gaubert et al., 2011) primers. PCRs were conducted in a total volume of 25 µl, containing 10 μl of the Ampliqon PCR Master Mix, 4 μl of DNA template (50 ng), and 1 μl of each primer. PCR amplification was initiated with a hot start phase and then followed 10 s of denaturation at 94°C, 40 cycles consisting of 60 s at 94°C (denaturation), 60 s at 55°C (primer annealing), and 90 s at 72°C (elongation), to end with a final elongation of 10 min at 72°C. Double‐strand cycle Sanger sequencing was performed using the BigDye Terminator Cycle Sequencing kit v.3.1 (Applied BioSystems), and electrophoresis of the purified sequencing product was carried out on an ABI PRISM 3730xl automatic sequencer. Sequences were edited for correction with SeqScape v.2.6 software (Applied Biosystems). All new sequences have been submitted to GenBank (accession numbers: MZ043127‐MZ043153).

#### 2.1.2 Phylogenetic analysis

Iranian sequences of *U. edwardsii* (24 sequences) and *U. auropunctata* (3 sequences), and sequences from GenBank, including eight sequences of *U. edwardsii,* six sequences of *U. auropunctata* as well as three sequences of *Urva javanica*, were aligned using the Clustal W algorithm (Thompson et al., 1994) implemented in Mega v.7 (Kumar et al., 2016), and final adjustments were made by eye. In addition, the sequence of *Herpestes brachyurus* (KY117547) was used as the outgroup. The HKY+ G was selected as the best‐fitting model of DNA substitution, using Bayesian information criterion (BIC) implemented in jModelTest (Posada, 2008). Alignments of noncoding regions of mtDNA frequently present gaps, and thus, gaps were treated as fifth base in the marker.

Bayesian phylogenetic analyses were carried out in MrBayes v.3.2 (Ronquist et al., 2012) with two independent runs of four Markov chains (one cold and three heated) over 10,000,000 generations and sampling every 1,000 generations. The first 25% of the sampled trees and estimated parameters were discarded as burn‐in. Convergence of the model parameters was monitored using the program Tracer v.1.7.1 (Rambaut, Drummond, Xie, Baele, & Suchard, 2018). The consensus phylogenetic tree was then edited in FigTree v.1.4.4 (http://tree.bio.ed.ac.uk/software/figtree/). Fixation index (*F*_ST_) was calculated using ARLEQUIN (Excoffier & Heckel, 2006), and a significance test of 10,000 permutations, of the obtained estimates of *F*_ST_, was performed. In addition, three methods for species delimitation were used to identify the specific boundaries in the species: (i) the Poisson tree process model (PTP; Zhang et al., 2013), (ii) the General Mixed Yule Coalescent method (GMYC) of Pons et al. (2006), and (iii) the Automated Barcode Gap Discovery (ABGD) method of Puillandre et al. (2012). These approaches were implemented on online server: ABGD (https://bioinfo.mnhn.fr/abi/public/abgd/), PTP (https://species.h‐its.org/ptp/), and GMYC (https://species.h‐its.org/gmyc/) with the default parameters.

The divergence time of the major clades was estimated using a Bayesian molecular clock approach implemented in the program BEAST v2.4.7 (Bouckaert et al., 2014). All molecular clocks need to be calibrated using independent information such as fossils. Fossils attributed to *Urva* are scarce (Peigné et al., 2005). The oldest specimen of the common mongoose of Asia found in Pakistan may be as old as 10 Mya, while the youngest is about 7 Mya (Barry, 1983). The Asian *Urva* diverged in the Early to Middle Miocene (ca. 15 Mya, Patou et al., 2009). During the period of 11–9 Mya, many divergence events occurred within the Asian mongooses with one branch leading to extant of the outgroup (*Herpestes brachyurus*) and another leading to the remaining of Asian mongoose (*U*. *edwardsii*, *U. javanicus,* and *U*. *auropunctatus*) (Patou et al., 2009). Given the datasets assembled here, we chose to use the minimum boundary for *H. brachyurus* and the remaining Asian mongoose (11–9 Mya) as the calibration point (lognormal distribution, M: 2.69, *SD*: 0.07). To account for lineage‐specific rate heterogeneity, a lognormal relaxed clock model was used and a Yule model of speciation was used for the tree prior. Two independent Markov chains were run for 3 x10^7^ generations, sampling every 1,000 generations was employed to construct a maximum clade credibility tree. The log parameters and tree files from each run were combined, using the LOGCOMBINER v.1.8. (Rambaut & Drummond, 2015), and convergence diagnostics were assessed using TRACER v.1.6 (Rambaut et al., 2007).

Measures of DNA polymorphism were estimated using DnaSP v.5 (Librado & Rozas, 2009), including the number of haplotypes (H), haplotype diversity (*h*), and nucleotide diversity (*π*) for each clade separately and the whole samples in Iran. A median‐joining (MJ) network was constructed for *U. edwardsii* using PopART v.1.7 (Leigh & Bryant, 2015) with the default settings.

#### 2.1.3 Ecological niche modeling

Species presence localities were compiled opportunistically from different sources including genetic specimens, camera‐trap detections, trappings, and environmental guards' sightings across the species range (Figure 1). The presence localities of Indian gray mongoose were explored on biodiversity datasets (i.e., VertNet, iDigBio, and Arctos). However, the presence points on these datasets was not included in the ESM analyses for two reasons: (1) There was a significant overlap among presence points on these datasets and those that we compiled for the analysis, considering 1‐km buffer around each point, and (2) the accuracy of the presence localities recorded in these databases was unknown, especially for countries with scarcity of data or studies that are conducted at a small scale.

We excluded the Indian gray mongoose occurrence localities in the contact zone of the two identified clades in the process of ecological niche modeling of clades as we did not have genetic samples from their regions, and therefore, their genetic clades were unknown. However, these localities were included in the ENM using all occupied localities clades together. Spatial autocorrelation in presence localities was assessed by Moran's I index for each variable separately in R package *raster* (Hijmans & van Etten, 2011). For variable with high and positive Moran's *I* value (Table S1) and to avoid bias in ENMs due to unequal sampling effort across the region, we spatially filtered presence localities and considered localities that were separated at least 1 km from each other. Finally, a total of 39 and 29 occurrence points for the Clade 1 and 2 were retained for modeling, respectively. To predict ecological niche divergence between the identified genetic clades of *U. edwardsii*, we adopted a between‐clade ENM based on climatic and nonclimatic environmental variables and a climate only niche comparison based on a PCA on climatic variables. For the first procedure, we used four categories of variables (land cover, anthropogenic, topographic, and climatic) within an ensemble modeling framework. Climatic data were obtained from 19 bioclimatic variables (CHELSA; http://chelsa‐climate.org) with spatial resolution of a 30 arc‐second. To account for multi‐collinearity among bioclimatic variables and avoid overparameterization of the models, we calculated Pearson's correlation tests among all pairs of variables and also variance inflation factor (VIF) for each variable in R package *sudm* (Naimi 2017). From each pair of highly correlated variables (*r* > 0.80), we removed those with higher VIF (>3) from further analysis. This reduction of variables resulted in the inclusion of eight climate predictors in the final ENM including temperature annual range, mean temperature of wettest quarter, mean temperature of warmest quarter, precipitation of wettest month, precipitation seasonality, precipitation of warmest quarter, and precipitation of coldest quarter. Four land use and land cover types were extracted from the Global Land Cover by National Mapping Organization (GLCNMO) (Kobayashi et al., 2017) including the herbaceous with sparse density of tree and shrub, sparse herbaceous, consolidated land, and unconsolidated land. We calculated the proportion of cells occupied by each type within a circle moving window with radius of 7 km in FRAGSTATS (McGarigal et al., 2002). To account for the effects of anthropogenic impacts on the occurrence of *U. edwardsii*, human footprint layer was downloaded from the 2009 Human Footprint (Venter et al., 2018). Elevation and topographic roughness were calculated from the Shuttle Radar Topography Mission (SRTM) elevation model (http://srtm.csi.cgiar.org).

To create a reliable map of highly suitable habitats for each clade considering the low number of occurrence localities, we adopted ensembles of small models’ (ESMs) (Breiner et al., 2015, 2018) in R packages *ecospat* (Broennimann et al., 2015) and *biomod* (Thuiller et al., 2021). ESMs represent a novel technique for predicting spatial niche of species with few observations (Breiner et al., 2018). By reducing the number of predictor variables and averaging simple small bivariate models to an ensemble, this approach avoids overfitting. For each clade, numerous bivariate maximum entropy models (MaxEnt) (Phillips et al., 2006) were calibrated and evaluated. Then, the final ESMs for each clade were calculated using the weighted average of the all resulting Somers’ D (i.e., rescaled AUC) values of the bivariate models. Models with a Somers’ D lower than 0 were not included in the ESMs. All bivariate models were calibrated with the threefold cross‐validation and 10,000 background points and were calibrated using the 70% of occurrence localities as training and 30% as evaluation data.

We calculated AUC and continuous Boyce index (Hirzel et al., 2006) to evaluate the performance of each model separately and overall ESM. All distribution analyses were conducted at a spatial resolution of 1 km, as a balance between obtaining sufficient spatial detail and avoiding excessive computational load and storage demands. In addition to running the ESMs for each genetic clade separately, we predicted the habitat suitability of *U. edwardsii* using all occupied localities of both clades together.

To compare climate niche utilized by two clades and quantify climate niche divergence/conservatism, we used the method developed by Broennimann et al. (2012). This method calculates density of occurrences and climate variables using a kernel density function in the multivariate PCA space and then calculated niche overlap and two randomization procedures (niche equivalency and niche similarity) to test the hypotheses of niche divergence/conservatism. This technique merges all occurrence localities in a pool and performs niche equivalency and niche similarity tests based on the whole dataset. We extracted seven above‐mentioned bioclimatic variables and calculated the orthogonal climatic axes, and depicted the position of the occurrence localities of *U. edwardsii* and climate niche space as a representative of their climate niche on climatic axes. The available climate niche space for each clade of *U. edwardsii* was defined as all pixels of the eight bioclimatic predictors within a buffer of 20‐km enclosing the species occurrence localities. The observed niche overlap score of the two clades across the gradients of the PCA space was computed based on a Schoener's D metric. Niche equivalency determines whether climate niche space of the clades is significantly different from those expected by chance. The pseudomodels are generated by randomly assigning identity to occurrence points. If the niche overlap value fell outside the 95% of the null hypotheses, the niche equivalency between clades is rejected (*p* < .05). Since niche equivalency test disregards surrounding space, we also performed niche similarity (background similarity) test between clades. Niche similarity test uses randomization to determine whether climate difference detected between clades can be explained by difference in the environmental background they occurred. In niche similarity test, random models are generated by using randomly generated occurrence points. The niche similarity test was carried out in both directions. The mean of overlap metrics obtained by 100 pseudoreplicates was compared to real overlap metric by empirical models. The null hypothesis of niche similarity test is rejected when the observed niche overlap value is significantly lower than the values generated by pseudoreplicates. Significant niche identity and background similarity indicates differences in the predicted climate niches of both clads is due to differences in the overall environment between regions. On the other hand, significant niche identity and insignificant background similarity indicate that the differences in the climate niches of clades are not due to underlying environmental differences and this difference is due to differences in the niche utilized by each clade in each region (May et al., 2011).

## 3 RESULTS

### 3.1 Phylogenetic analysis and genetic structure

**FIGURE 2:**
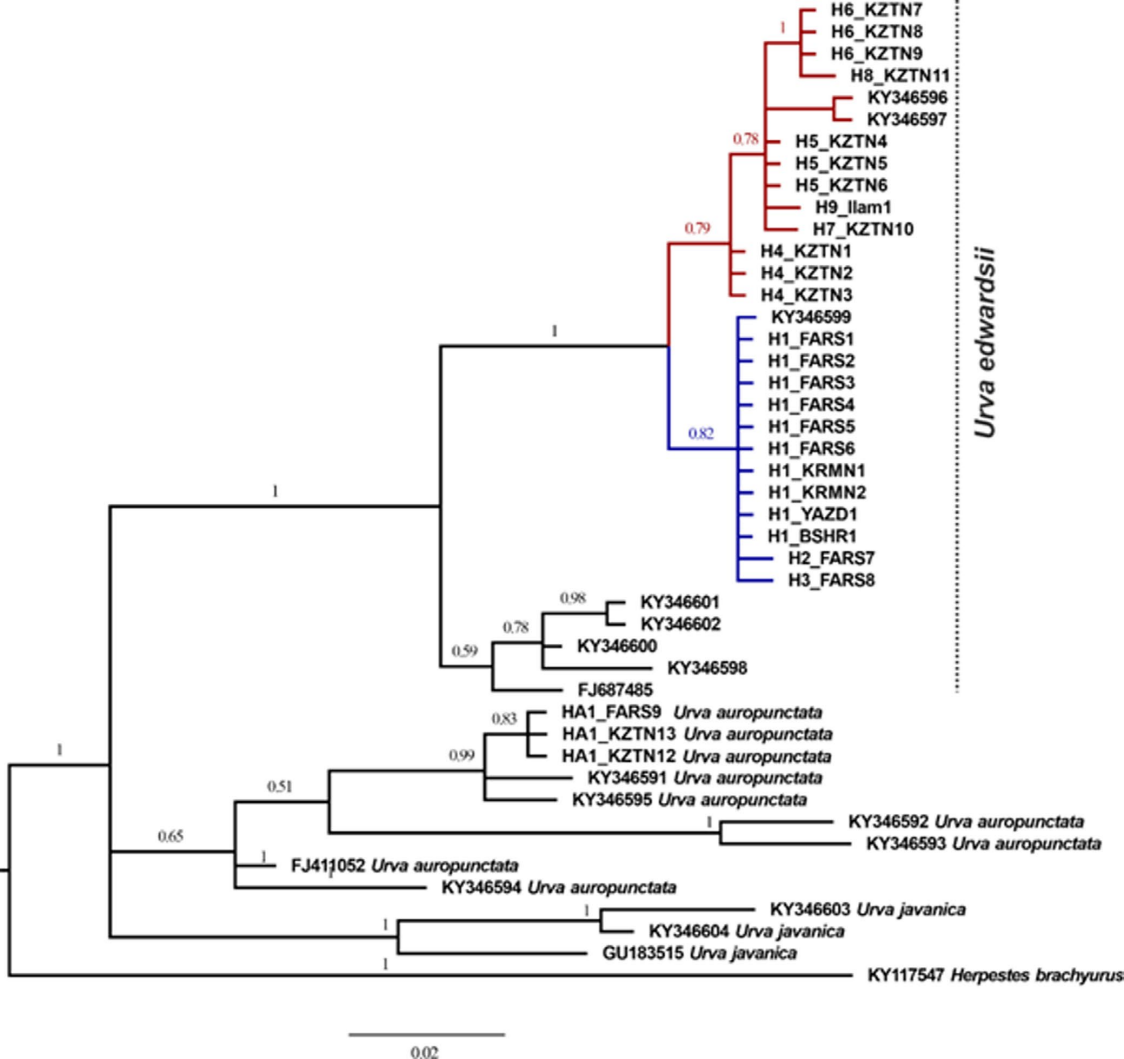
Phylogeny of *Urva edwardsii* (Clade 1, blue, and Clade 2, red) and *Urva auropunctata* from Bayesian analysis of CR gene sequences. The trees were summarized with the majority‐rule consensus tree. Numbers on nodes are Bayesian posterior probabilities

**FIGURE 3:**
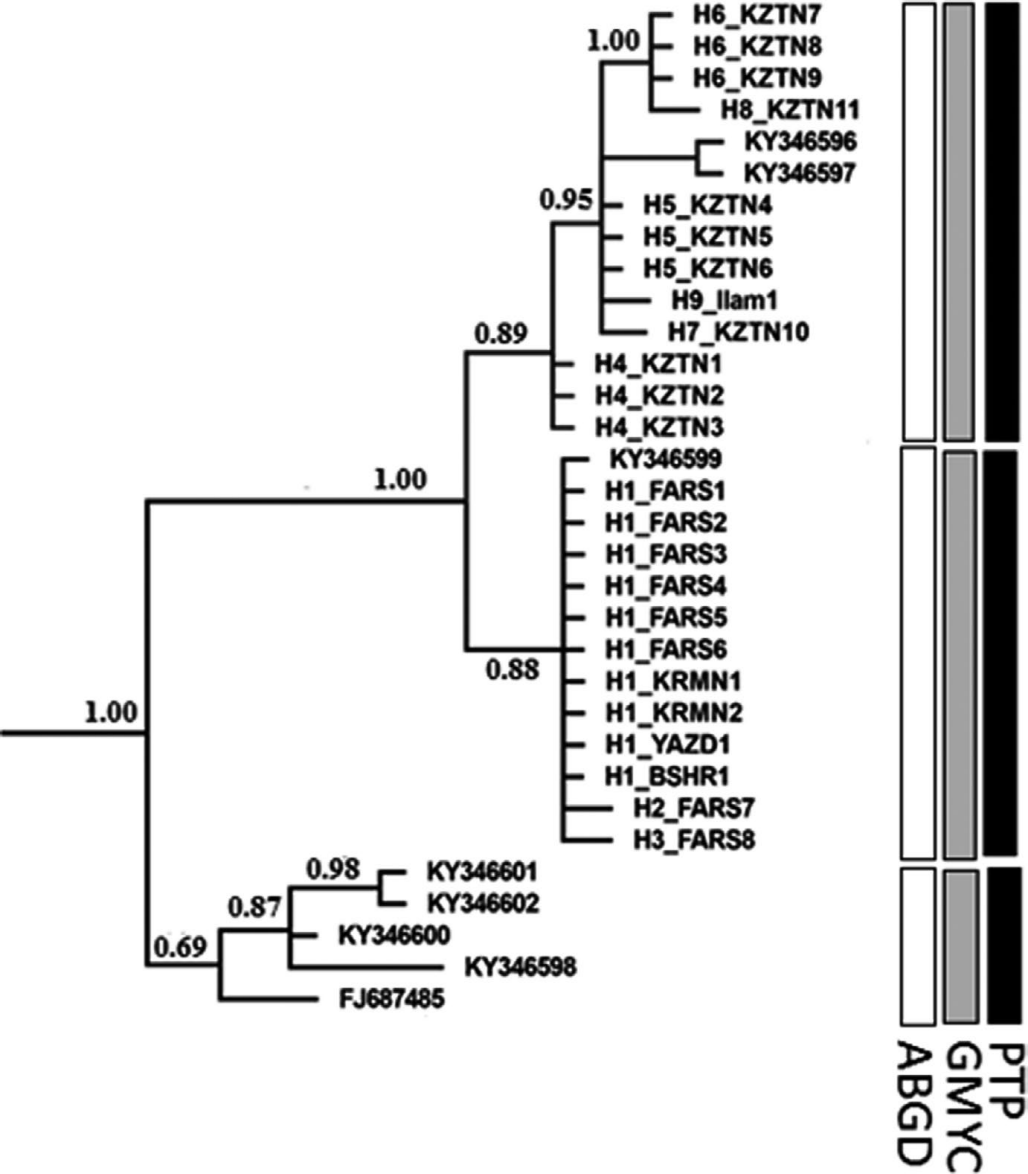
Species delimitation analyses based on the control region sequences of *Urva edwardsii*, using Automatic Barcode Gap Discovery (ABGD), Poisson Tree Processes (PTP), and General Mixed Yule Coalescent (GMYC) methods. Bayesian posterior probability are given on the branches

PCR amplifications of the control region were entirely successful for all 24 specimens of *U. edwardsii* and three specimens of *U. auropunctata* (yielding fragments of 516 bp) collected across the species range in Iran. The Bayesian inference consensus tree with posterior probabilities (PP) showed that the Indian gray mongoose in Iran has divided into two clades corresponding to the haplotypes of the species range in south and southeast, hereafter Clade 1 (blue color; Figure 2) and another clade corresponding to the rest of the species range in southwest, hereafter Clade 2 (red color; Figure 2). While sequences from Bangladesh (KY346600‐KY346602), UAE (KY346598), and Italy (FJ687485) separated as a different clade from Iranian sequences (PP = 1), one sequence from UAE (KY346599) and two sequences from Bahrain (KY346596 and KY346597) placed in Iranian clades of 1 and 2, respectively. The fixation index between the two Iranian clades was 0.31. The *F*_ST_ between Clade 1 and Clade 3 (sequences from Bangladesh, UAE, and Italy) was 0.57. Pairwise *F*_ST_ between clades 2 and 3 was 0.45. All estimates were significant at *p* < .05 based on 10,000 permutations with Bonferroni corrections (Rice, 1989). Results of ABGD, PTP, and GMYC obtained with control region sequences are summarized in Figure 3. The three groups (clades 1, 2, and 3) were supported by all three species delimitation methods (GMYC, ABGD, and PTP).

The divergence time for the two Iranian clades (clades 1, 2) was averaged at about 4.9 Mya with 95% highest posterior density (HPD) intervals of 3.3–6.5. The divergence time of clade 3 (sequences from Bangladesh, UAE, and Italy) and the lineage leading to Iranian clades (1, 2) was estimated at about 6.0 Mya (95% HPD: 5.2–7).

**FIGURE 4:**
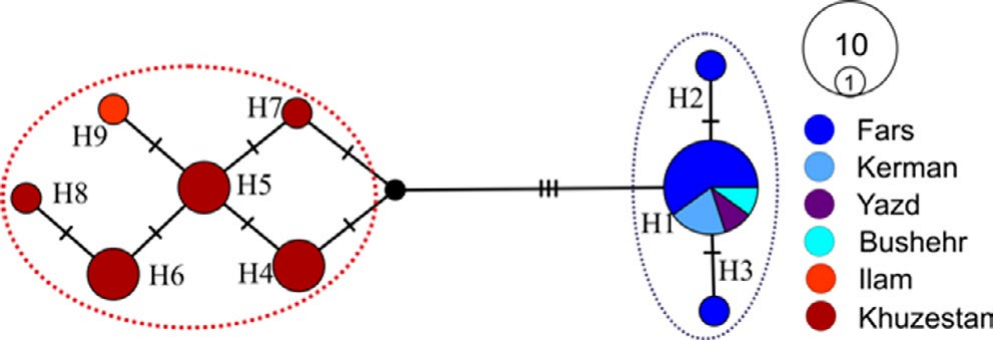
Median‐joining network of CR sequences of *Urva edwardsii* in Iran. Mutational steps among haplotypes are signaled with dash lines, and small, filled black circles refer to inferred missing haplotypes. Each circle represents a different haplotype, whereby areas of circles are proportional to the number of sampled individuals (see the legend for the circle sizes of one and ten samples, respectively). Blue and red dash lines comprise two clades belonging to the *Urva edwardsii* in south–southeast (Clade 1) and southwest (Clade 2) of Iran

The haplotype network provided further information regarding the haplotypes (Figure 4). Haplotype 1 was the most widespread haplotype of *U. edwardsii* and was found within 10 individuals in Clade 1 in four provinces of Fars, Kerman, Yazd, and Bushehr. Haplotype network showed divergence between the Indian gray mongooses in Iran. In line with the results obtained from the phylogeny tree (Figure 2), the haplotypes derived from the *U. edwardsii* were divided into two separate groups (i.e., clades 1 and 2).

Only one unique haplotype was detected among three sequences of *U. auropunctata*, but nine unique haplotypes were detected among the sequences generated for CR of *U. edwardsii* with an overall haplotype and the nucleotide diversity *h* = 0.804 and *π* = 0.00627, respectively (Table 2). The haplotypes and nucleotide diversity were higher for Clade 2 (*h* = 0.864 and *π* = 0.00272) than Clade 1 (*h* = 0.318 and *π* = 0.00065) indicating a high level of genetic variation in Clade 1 compared to Clade 2.

**TABLE 2:** Genetic diversity parameters of *Urva edwardsii* in Iran

Species	Population	*N*	*S*	*H*	*h*	*π*
*U. edwardsii*	Clade 1	12	2	3	0.318 ± 0.164	0.00065 ± 0.00036
	Clade 2	12	5	6	0.864 ± 0.064	0.00272 ± 0.00043
	Total	24	10	9	0.804 ± 0.069	0.00627 ± 0.0005

Note

*N*, number of individuals; *S*, number of segregating sites, *H*, number of haplotypes; *h*, haplotype diversity; *π*, nucleotide diversity.

### 3.2 Ecological niche modeling

Ensembles of small models performed reasonably well in predicting suitable habitats of both clades of *U. edwardsii*. The ESMs produced fairly high AUC and Boyce index values for predicting the ecological niche of Clade 1 (AUC = 0.940, Boyce index = 0.926) and Clade 2 (AUC = 0.978, Boyce = 0.812). The results of ESMs showed that the five most important variables for predicting habitat suitability of *U. edwardsii* in Clade 1 were precipitation seasonality, human footprint, sparse herbaceous, consolidated land, and herbaceous with sparse density of tree and shrub. On the other hand, the EMS predicted that suitable habitats of *U. edwardsii* in Clade 2 were related to mean temperature of warmest quarter, precipitation of coldest quarter, herbaceous with sparse density of tree and shrub, human footprint, and elevation (Table 3).

**TABLE 3:** The estimate results of the percent contributions of the variables in predicting ecological niche of *Urva edwardsii* using ensemble of small models

Climate variable	% Contribution for the Clade 1	% Contribution for the Clade 2	% Contribution for both clades
Temperature Annual Range (Bio7)	0.20	2.50	0.10
Mean Temperature of Wettest Quarter (Bio8)	1.3	0.00	0.50
Mean Temperature of Warmest Quarter (Bio10)	0.80	34.00	5.60
Precipitation of Wettest Month (Bio13)	0.40	0.30	0.30
Precipitation Seasonality (Bio15)	33.2	0.10	9.70
Precipitation of Warmest Quarter (Bio18)	0.90	8.60	12.10
Precipitation of Coldest Quarter (Bio19)	1.5	13.90	19.50
Herbaceous with sparse density of tree and shrub	5.1	11.80	3.80
Sparse herbaceous	13.5	0.70	2.90
Consolidated land	5.5	2.40	3.80
Unconsolidated land	1.3	6.30	1.30
Human footprint	35.5	10.20	35.50
Elevation	0.10	8.70	3.90
Topographic roughness	0.50	0.50	1.00

**FIGURE 5:**
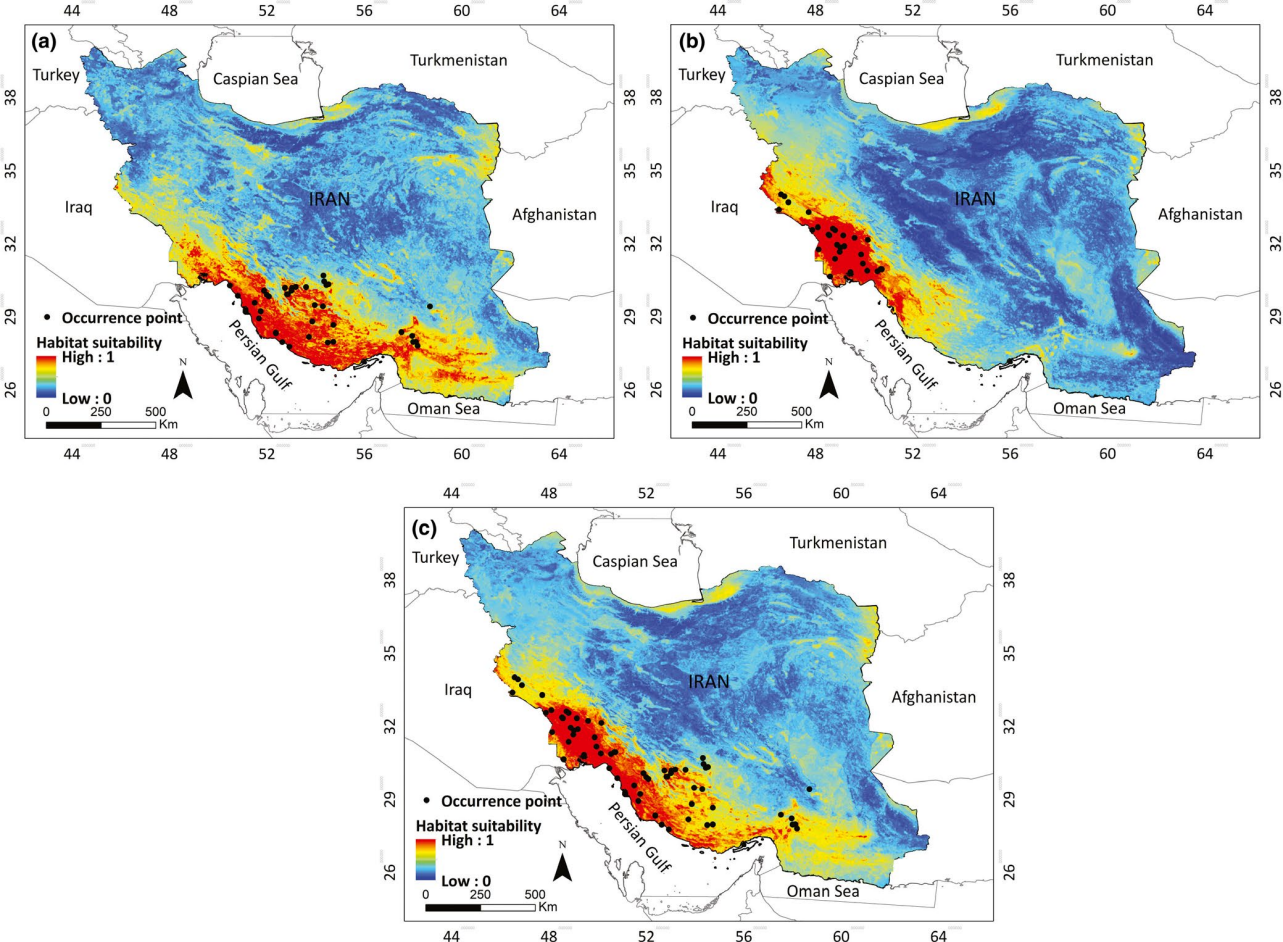
Predicted habitat suitability of *Urva edwardsii* based on presence localities in Clade 1 (a), Clade 2 (b), and all occupied localities of both clades (c) obtained with ESMs framework. Higher and lower probability of occurrence is indicated by red and blue color, respectively

The ESMs showed a notable consistency in predicting habitat suitability of both clades when compared with occurrence records. The potential distribution of *U. edwardsii* for Clade 1 is almost two times larger than the genetic Clade 2 (Figure 5). The relative habitat suitability values predicted by the ESM for the *U. edwardsii* in both clades revealed their niche separation (Figure 5). The suitable habitats for Clade 2 is predicted to be concentrated in west and southwestern Iran, whereas the predicted distribution for the Clade 1 showed a wider distribution and is distributed to south and southeast of Iran.

Between‐clade climate only niche comparison based on PCA revealed distinct climate niche between two genetic clades. The first two components (PC1 and PC2) explained 40.30% and 29.80% of the variation in climatic variables, respectively. PC1 was mostly correlated with temperature‐related factors (e.g., mean temperature of wettest quarter, temperature annual range, and mean temperature of warmest quarter), while PC2 was highly correlated with precipitation‐related factors (precipitation of coldest quarter, and precipitation of wettest month; Table 4).

**TABLE 4:** Summary of the PCA on the eight climate variables extracted from the area defined by a 20‐km buffer around presence localities of *Urva edwardsii*

Climatic variables	PC1	PC2	PC3	PC4
Temperature annual range (Bio7)	0.47	0.23	0.30	−0.42
Mean temperature of wettest quarter (Bio8)	−0.52	0.27	0.06	−0.31
Mean temperature of warmest quarter (Bio10)	−0.36	0.47	0.14	−0.40
Precipitation of wettest month (Bio13)	0.30	0.48	−0.42	−0.09
Precipitation seasonality (Bio15)	−0.33	0.06	−0.69	0.12
Precipitation of warmest quarter (Bio18)	0.21	−0.41	−0.44	−0.69
Precipitation of coldest quarter (Bio19)	0.37	0.48	−0.20	0.28
Eigenvalue	14.05	10.39	6.53	2.65
Explained variance (%)	40.30	29.80	18.73	7.59

**FIGURE 6:**
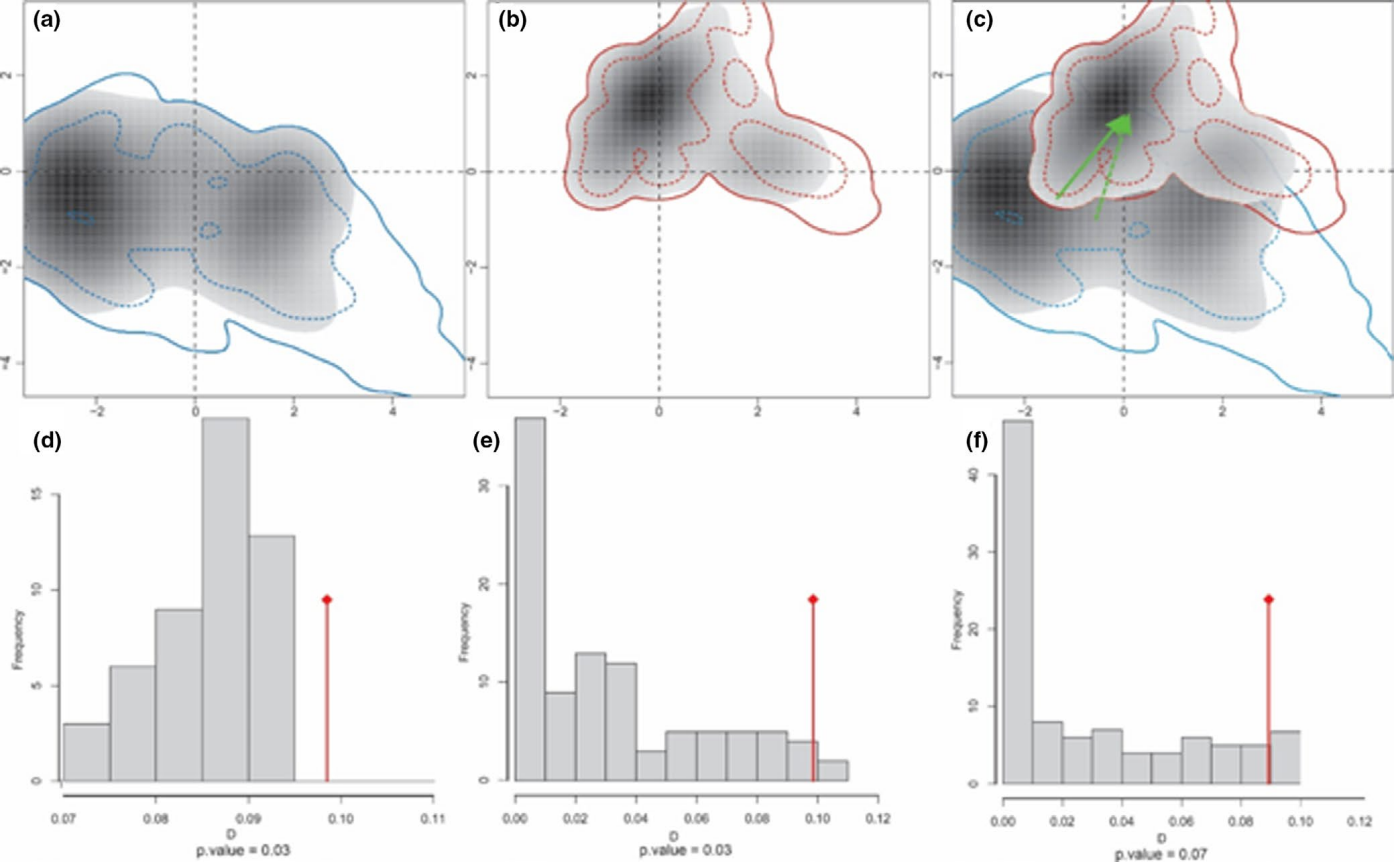
Climate niche of Indian gray mongooses along the first two of axes of the PCA in the south and southeast (a) and southwest (b) of Iran. Panel (c) represents the degree of niche overlap between two clades. The green arrow shows how the center of the niche has changed between the clades. Transparent shading indicates density of the occurrence localities of each clade per cell, and solid and dashed lines show 100% and 50% of the background environment space, respectively. Histograms (d–f) show the results of niche equivalency test (d) and niche similarity of Clade 1 to Clade 2 (e) and niche similarity Clade 2 to Clade 1 (f). Vertical red lines and gray bars show the observed and simulated niche overlap D between the two clades, respectively

The comparison of climatic niches of both clades along the PC1 and PC2 showed low climate niche overlap (*D* = 0.256). The relatively low niche overlap suggested that two genetic clades of *U. edwardsii* occupy considerably distinct climate niches. Considering niche position of both clades on climatic axes (Figure 6), Indian gray mongooses in the Clade 1 occupy larger climatic niche than those in the Clade 2. The niche equivalency test highlighted that the observed climate niches were significantly different than those expected by chance (*p* < .05) confirming that the niches of the identified clades of *U*. *edwardsii* are significantly distinct. The background test comparing the clade in southwestern Iran (Clade 2) occurrences with the background space of the clade in south and southeast (Clade 1) revealed that the observed value of niche similarity is lower than expected under the null hypothesis. However, when comparing in the opposite direction, the observed overlap is higher (a more similar ecological niche), yet not significant. Randomization tests (i.e., niche identity and background similarity) showed that environmental space occupied by clades is similar to some extent, but the niches that they utilize (identity test) differ between the distribution ranges of clades.

## 4 DISCUSSION

The present study was the first to employ a combination of genetic data and ecological niche models to evaluate intraspecific genetic and ecological variation within a less studied carnivore, the Indian gray mongoose. Our findings provide evidence in supporting the idea that incorporating ecological niche models into phylogeographic analyses would improve the accuracy of intraspecific genetic information and predictive ability to population boundaries. The adopted methodology has great implications for conservation unit assessment for the species and can provide valuable information regarding the feasibility of this approach to many species around the world. We showed two main results, which are elaborated in the sections below. First, phylogenetic analysis suggested the existence of two genetic clades within the *U. edwardsii* populations in Iran, one in southwest and the other in south and southeast. Second, a genetic clades‐specific combination of environmental variables among the clades was found, indicating the niches that each *U. edwardsii* clade utilizes differ between the distribution ranges of clades.

### 4.1 Genetic diversity and phylogenetic clusters of *U. edwardsii*

The result of phylogenetic tree showed that *U. edwardsii* is separated from *U. auropunctata*, which is in line with the results of Veron and Jennings (2017). It also showed the existence of different clades for *U*. *edwardsii* in Iran. Based on the phylogenetic tree (1995 bp) from a combined dataset of Cyt*b* + ND2 + FGB (Veron & Jennings, 2017), a specimen from Sumatra (UeB34042) forms a paraphyletic group to all other specimens, while Middle Eastern specimens placed into the two clades, including Bahrain (UeTC144‐UeTC145), UAE (UeTC147‐UeTC148), and Iran (UeTC149) in one, and specimens from Bangladesh (UeTC294‐UeTC296), UAE (UeTC146), and Netherland (UeTC293) to another. Also, one specimen from Nepal (UeA163188) is placed as a sister group to the two Middle Eastern clades (see Veron and Jennings (2017) for accession numbers). Here, available CR sequences (Veron & Jennings, 2017), that is, UeTC294‐UeTC296, UeTC144‐UeTC146, UeTC148, and UeFj678485 were aligned with sequences of current study to reconstruct the phylogenetic tree. The CR phylogenetic tree and species delimitation approaches showed existence of three clades, including Middle Eastern 1 (Clade 1), Middle Eastern 2 (Clade 2), and Asiatic (other). The interesting result was the splitting of the Iranian *U. edwardsii* populations into two distinct clades, Clade 1 at the south–southeastern and Clade 2 at the southwestern Iran. Restricted gene flow or adaptation to different ecological conditions in allopatric or parapatric populations would have resulted in phenotypic divergences, genetic difference, and accelerating the evolution of reproductive isolation (Kozak et al., 2008; May et al., 2011; Ruiz‐Sanchez & Sosa, 2010). Both biotic and abiotic factors are involved in determining spatial patterns of the species' genetic variation and promoting adaptive divergence and speciation (Peterson et al., 1999; Rissler & Apodaca, 2007).

The estimated divergence time between the two Iranian clades (4.9 Mya) coincides with the uplifting of the Zagros Mountains during the Early Pliocene. The uplifting of Zagros Mountains began as a result of the Arabian plate impinging on Eurasia in the Late Miocene (Salah, 2019; Sborshchikov et al., 1981). The separation of the Arabian plate from Africa was accelerated in the beginning of the Pliocene (ca. 5 Mya) (Girdler, 1984) and led to mountain‐building on the western margins of the Iranian Plateau (Macey et al., 1998).

Based on our analysis, this barrier seems to have prevented the distribution of *U. edwardsii* populations between the western and eastern parts of the mountains as a result of vicariance events. The Clade 2 is mainly located in the Khuzestan Plain, which is a relatively flat region of Iran, isolated from the Clade 1 by the southern Zagros Mountains. Khuzestan comprises a southeastern extension of the Mesopotamian plain and includes part of the forested Zagros Mountains. The Zagros mountain‐building event, as a barrier to gene flow, has been also reported in some taxonomic groups, in particular, for small mammals (Asadi‐ Aghbolaghi et al., 2019; Rezazadeh et al., 2020).

The two genetic clades of *U. edwardsii* in eastern (mostly related to Khuzestan Plain) and western parts (related to the southern part of Zagros mountain range) of its distribution in Iran can be considered as two conservation units according to the genetic and ecological concept. Further investigation is needed into the possible presence of a cryptic subspecies.

### 4.2 Different ecological niches of *U. edwardsii* clades

There are potentially genetic differences between the two regions based on mtDNA, and that the two regions have different environmental characteristics, which may lead to different adaptations. Although ecological niche models are often used to map distribution range of threatened species and evaluate environmental factors affecting them (e.g., Ahmadi et al., 2017; Khosravi et al., 2019; Shahnaseri et al., 2019), hypothesis regarding ecological exchangeability (May et al., 2011), niche segregation between species, subspecies, or populations of same species in different regions or seasons, and intraspecific or interspecific taxonomic distinction (Nakazato et al., 2010) can be tested using these noninvasive and multifunctional tools. We performed niche differentiation tests to identify the environmental constraints for the distribution of *U. edwardsii* and explore the hypothesis that adaptation to different environmental condition are contributing to genetic structuring in Indian gray mongoose. In accordance to genetic findings, our ecological niche models showed little geographic overlap between two genetic clades of *U*. *edwardsii*, indicating that clades differ in their niches and occupy a part of the geographic range in south of Iran where environmental conditions are suitable (Figure 5). Also, our environmental niche model showed that ensemble of small models of each genetic clade alone (Figure 5a and b) was better than models of two genetic clades together (Figure 5c). However, the distribution models based on the all presence localities cover areas highly suitable for both genetic clades (Figure 5c), and the ESMs of the two clades separately showed that Clade 1 is commonly found at localities that are less suitable for Clade 2 and vice versa.

Although the distribution of both clade appears to be highly dependent on the herbaceous with sparse density of tree and shrub (Table 3), climatic and nonclimatic environmental variables contributed differently to each clade climate niche: “precipitation seasonality” and “human footprint” for the Indian gray mongooses in south–southeast and “mean temperature of warmest quarter” and “precipitation of coldest quarter” for the Indian gray mongooses in southwest of Iran. It is plausible that human footprint is important variable that could determine the establishment of *U. edwardsii* across the species distribution range in Iran, while the differences in other variables could be acting as factors of differentiation between regions. However, *U. edwardsii* in southwest of Iran have smaller niche breadth and lives in areas where precipitation of warmest season (summer) is relatively more than south and southeast of Iran, and *U. edwardsii* in south and southeast of Iran inhabits areas with dry summers. Thus, our results highlighted the importance of precipitation and temperature in providing climatically suitable niche for Indian gray mongoose in Iran.

Randomization tests confirmed that two genetic clades of *U. edwardsii* occupy distinct ecological niches that are similar, but undoubtedly not equivalent. The results of the niche identity test suggested a lack of ecological exchangeability confirming previous researches that morphological similarity does not necessarily equate to ecological equivalence (Scriven et al., 2016).

## 5 CONCLUSION

In conclusion, our results showed that two genetic clades of Indian gray mongoose occupy distinct environmental spaces that are similar, but not equivalent, relatively small niche overlap between these clades. These findings show that two clades of *U. edwardsii* underwent significant changes of their range in environmental niche space during the process of occupation of region within their present distribution. The estimated divergence time between the two Iranian clades (4.9 Mya) coincides with the uplifting of the Zagros Mountains during the Early Pliocene. The Zagros mountain‐building event seems to have prevented the distribution of *U. edwardsii* populations between the western and eastern parts of the mountains as a result of vicariance events.

### 5.1 Conservation implications

Our predicted ecological niche maps broadly estimate the most important ecological requirements that the species may need to establish a habitat. Also, the obtaining distribution data are informative to establish conservation priority and conservation efforts. As our genetic data confirmed the genetic structure among the populations of *U*. *edwardsii*, thus, a conservation status assessment should be conducted for both clades separately and may result in a subsequent definition of cryptic subspecies and conservation efforts. Also, it is important that conservation actions be taken in the immediate future to conserve the genetic variation of population in each region before it declines. Despite that the impact on native fauna of introduced Indian gray mongooses is not known, further introductions of any mongoose species to act as a biological control outside their native range are not recommended.

## CONFLICT OF INTEREST

The authors declare that they have no competing interests.

## AUTHOR CONTRIBUTIONS

**Razieh Oboudi:** Conceptualization (equal); Data curation (equal); Methodology (equal); Resources (equal). **Mansoureh Malekian:** Conceptualization (lead); Formal analysis (equal); Supervision (lead); Writing‐original draft (equal); Writing‐review & editing (equal). **Rasoul Khosravi:** Formal analysis (equal); Methodology (equal); Software (equal); Validation (equal); Writing‐original draft (equal); Writing‐review & editing (equal). **Davoud Fadakar:** Formal analysis (equal); Methodology (equal); Software (equal). **Mohammad Ali Adibi:** Data curation (equal).

## Supporting information

Table S1

## Data Availability

DNA sequences have been deposited in GenBank (https://www.ncbi.nlm.nih.gov/genbank/) under the accession no. MZ043127‐MZ043153.

## References

[ece38168-bib-0001] Ahmadi, M. , Nezami Balouchi, B. , Jowkar, H. , Hemami, M. R. , Fadakar, D. , Malakouti‐Khah, S. , & Ostrowski, S. (2017). Combining landscape suitability and habitat connectivity to conserve the last surviving population of cheetah in Asia. Diversity and Distributions, 23(6), 592–603. 10.1111/ddi.12560

[ece38168-bib-0002] Alvarado‐Serrano, D. F. , & Knowles, L. L. (2014). Ecological niche models in phylogeographic studies: Applications, advances and precautions. Molecular Ecology Resources, 14(2), 233–248. 10.1111/1755-0998.12184 24119244

[ece38168-bib-0003] Asadi‐ Aghbolaghi, M. , Ahmadzadeh, F. , Kiabi, B. , & Keyghobadi, N. (2019). The permanent inhabitant of the oak trees: Phylogeography and genetic structure of the Persian squirrel (*Sciurus anomalus*). Biological Journal of the Linnean Society, 127, 197–212. 10.1093/biolinnean/blz032

[ece38168-bib-0004] Barry, J. C. (1983). *Herpestes* (Viverridae, Carnivora) from the Miocene of Pakistan. Journal of Paleontology, 57(1_Part_I), 150–156.

[ece38168-bib-0005] Barton, N. (2008). The effect of a barrier to gene flow on patterns of geographic variation. Genetics Research, 90(1), 139–149. 10.1017/S0016672307009081 18289408

[ece38168-bib-0006] Barun, A. , Niemiller, M. L. , Fitzpatrick, B. M. , Fordyce, J. A. , & Simberloff, D. (2013). Can genetic data confirm or refute historical records? The island invasion of the small Indian mongoose (*Herpestes auropunctatus*). Biological Invasions, 15, 2243–2251. 10.1007/s10530-013-0447-6

[ece38168-bib-0007] Bennett, C. , Pastorini, J. , Dollar, L. , & Hahn, W. (2009). Phylogeography of the Malagasy ring‐tailed mongoose, Galidia elegans, from mtDNA sequence analysis. Mitochondrial DNA, 20, 7–14. 10.1080/19401730802699006. 19565675

[ece38168-bib-0008] Bouckaert, R. , Heled, J. , Kühnert, D. , Vaughan, T. , Wu, C.‐H. , Xie, D. , Suchard, M. A. , Rambaut, A. , & Drummond, A. J. (2014). BEAST 2: A software platform for Bayesian evolutionary analysis. PLOS Computational Biology, 10(4), e1003537. 10.1371/journal.pcbi.1003537 24722319 PMC3985171

[ece38168-bib-0009] Bradburd, G. S. , Ralph, P. L. , & Coop, G. M. (2013). Disentangling the effects of geographic and ecological isolation on genetic differentiation. Evolution, 67(11), 3258–3273. 10.1111/evo.12193 24102455 PMC3808528

[ece38168-bib-0010] Breiner, F. T. , Guisan, A. , Bergamini, A. , & Nobis, M. P. (2015). Overcoming limitations of modelling rare species by using ensembles of small models. Methods in Ecology and Evolution, 6(10), 1210–1218. 10.1111/2041-210X.12403

[ece38168-bib-0011] Breiner, F. T. , Nobis, M. P. , Bergamini, A. , & Guisan, A. (2018). Optimizing ensembles of small models for predicting the distribution of species with few occurrences. Methods in Ecology and Evolution, 9(4), 802–808. 10.1111/2041-210X.12957

[ece38168-bib-0012] Broennimann, O. , Di Cola, V. , & Guisan, A. (2015). ecospat: Spatial ecology miscellaneous methods. R package version 1. Retrieved from https://cran.r‐project.org/web/packages/ecospat/index.html

[ece38168-bib-0013] Broennimann, O. , Fitzpatrick, M. C. , Pearman, P. B. , Petitpierre, B. , Pellissier, L. , Yoccoz, N. G. , Thuiller, W. , Fortin, M.‐J. , Randin, C. , Zimmermann, N. E. , Graham, C. H. , & Guisan, A. (2012). Measuring ecological niche overlap from occurrence and spatial environmental data. Global Ecology and Biogeography, 21(4), 481–497. 10.1111/j.1466-8238.2011.00698.x

[ece38168-bib-0014] Chang, J. , Chen, D. E. , Ye, X. , Li, S. , Liang, W. , Zhang, Z. , & Li, M. (2012). Coupling genetic and species distribution models to examine the response of the Hainan partridge (Arborophila ardens) to Late Quaternary climate. PLoS One, 7(11), e50286. 10.1371/journal.pone.0050286 23185599 PMC3501459

[ece38168-bib-0015] Crandall, K. A. , Bininda‐Emonds, O. R. , Mace, G. M. , & Wayne, R. K. (2000). Considering evolutionary processes in conservation biology. Trends in Ecology & Evolution, 15(7), 290–295. 10.1016/S0169-5347(00)01876-0 10856956

[ece38168-bib-0016] Excoffier, L. , & Heckel, G. (2006). Computer programs for population genetics data analysis: A survival guide. Nature Reviews Genetics, 7(10), 745–758. 10.1038/nrg1904 16924258

[ece38168-bib-0017] Gaubert, P. , Machordom, A. , Morales, A. , López‐Bao, J. V. , Veron, G. , Amin, M. , Barros, T. , Basuony, M. , Djagoun, C. A. M. S. , San, E. D. L. , Fonseca, C. , Geffen, E. , Ozkurt, S. O. , Cruaud, C. , Couloux, A. , & Palomares, F. (2011). Comparative phylogeography of two African carnivorans presumably introduced into Europe: Disentangling natural versus human‐mediated dispersal across the Strait of Gibraltar. Journal of Biogeography, 38(2), 341–358. 10.1111/j.1365-2699.2010.02406.x

[ece38168-bib-0018] Gaubert, P. , & Zenatello, M. (2009). Ancient DNA perspective on the failed introduction of mongooses in Italy during the XXth century. Journal of Zoology, 279(3), 262–269. 10.1111/j.1469-7998.2009.00614.x

[ece38168-bib-0019] Ghaedi, Z. , Badri, S. , Saberi‐Pirooz, R. , Vaissi, S. , Javidkar, M. , & Ahmadzadeh, F. (2020). The Zagros Mountains acting as a natural barrier to gene flow in the Middle East: More evidence from evolutionary history of spiny‐tailed lizards (Uromasticinae: Saara). Zoological Journal of the Linnean Society, 192(4), 1123–1136. 10.1093/zoolinnean/zlaa113

[ece38168-bib-0020] Girdler, R. W. (1984). The evolution of the Gulf of Aden and Red Sea in space and time. Deep Sea Research Part A. Oceanographic Research Papers, 31, 747–762. 10.1016/0198-0149(84)90039-6

[ece38168-bib-0021] Gotelli, N. J. , & Stanton‐Geddes, J. (2015). Climate change, genetic markers and species distribution modelling. Journal of Biogeography, 42(9), 1577–1585. 10.1111/jbi.12562

[ece38168-bib-0022] Grady, K. C. , Ferrier, S. M. , Kolb, T. E. , Hart, S. C. , Allan, G. J. , & Whitham, T. G. (2011). Genetic variation in productivity of foundation riparian species at the edge of their distribution: Implications for restoration and assisted migration in a warming climate. Global Change Biology, 17(12), 3724–3735. 10.1111/j.1365-2486.2011.02524.x

[ece38168-bib-0023] Grady, K. C. , Laughlin, D. C. , Ferrier, S. M. , Kolb, T. E. , Hart, S. C. , Allan, G. J. , & Whitham, T. G. (2013). Conservative leaf economic traits correlate with fast growth of genotypes of a foundation riparian species near the thermal maximum extent of its geographic range. Functional Ecology, 27(2), 428–438. 10.1111/1365-2435.12060

[ece38168-bib-0024] Guisan, A. , & Thuiller, W. (2005). Predicting species distribution: Offering more than simple habitat models. Ecology Letters, 8(9), 993–1009. 10.1111/j.1461-0248.2005.00792.x 34517687

[ece38168-bib-0025] Heshmati, G. (2012). Vegetation characteristics of four ecological zones of Iran. International Journal of Plant Production, 1(2), 215–224.

[ece38168-bib-0026] Hijmans, R. , & van Etten, J. (2011). raster: Geographic analysis and modeling with raster data R package version 1.9‐33. https://CRAN.R‐project.org/package=raster

[ece38168-bib-0027] Hirzel, A. H. , Le Lay, G. , Helfer, V. , Randin, C. , & Guisan, A. (2006). Evaluating the ability of habitat suitability models to predict species presences. Ecological Modelling, 199(2), 142–152. 10.1016/j.ecolmodel.2006.05.017

[ece38168-bib-0028] Holt, B. , Lessard, J‐P. , Borregaard, M. , Fritz, S. , Araújo, M. , Dimitrov, D. , Fabre, P‐H. , Graham, C. , Graves, G. R. , Jønsson, K. , Nogués‐Bravo, D. , Wang, Z. , Whittaker, R. , Fjeldså, J. , & Rahbek, C. (2013). An update of Wallace’s zoogeographic regions of the world. Science, 339, 74–78. 10.1126/science.1228282 23258408

[ece38168-bib-0029] Ikeda, D. H. , Max, T. L. , Allan, G. J. , Lau, M. K. , Shuster, S. M. , & Whitham, T. G. (2017). Genetically informed ecological niche models improve climate change predictions. Global Change Biology, 23(1), 164–176. 10.1111/gcb.13470 27543682

[ece38168-bib-0030] Kalle, R. , Ramesh, T. , Sankar, K. , & Qureshi, Q. (2012). Diet of mongoose in mudumalai tiger reserve, southern India. Journal of Scientific Transactions in Environment and Technovation, 6, 44–51.

[ece38168-bib-0031] Karami, M. , Ghadirian, T. , & Faizolahi, K. (2016). The atlas of mammals of Iran. Jahad Daneshgahi.

[ece38168-bib-0032] Khosravi, R. , Hemami, M.‐R. , & Cushman, S. A. (2019). Multi‐scale niche modeling of three sympatric felids of conservation importance in central Iran. Landscape Ecology, 34(10), 2451–2467. 10.1007/s10980-019-00900-0

[ece38168-bib-0033] Knowles, L. L. , Carstens, B. C. , & Keat, M. L. (2007). Coupling genetic and ecological‐niche models to examine how past population distributions contribute to divergence. Current Biology, 17(11), 940–946. 10.1016/j.cub.2007.04.033 17475496

[ece38168-bib-0034] Kobayashi, T. , Tateishi, R. , Alsaaideh, B. , Sharma, R. C. , Wakaizumi, T. , Miyamoto, D. , Bai, X. , Long, B. D. , Gegentana, G. , Maitiniyazi, A. , Cahyana, D. , Haireti, A. , Morifuji, Y. , Abake, G. , Pratama, R. , Zhang, N. , Alifu, Z. , Shirahata, T. , Mi, L. , … Phong, D. X. (2017). Production of Global Land Cover Data – GLCNMO2013. Journal of Geography and Geology, 9, 1–15. 10.5539/jgg.v9n3p1

[ece38168-bib-0035] Kozak, K. H. , Graham, C. H. , & Wiens, J. J. (2008). Integrating GIS‐based environmental data into evolutionary biology. Trends in Ecology & Evolution, 23(3), 141–148. 10.1016/j.tree.2008.02.001 18291557

[ece38168-bib-0036] Kumar, S. , Stecher, G. , & Tamura, K. (2016). MEGA7: Molecular evolutionary genetics analysis version 7.0 for bigger datasets. Molecular Biology and Evolution, 33(7), 1870–1874. 10.1093/molbev/msw054 27004904 PMC8210823

[ece38168-bib-0037] Leigh, J. W. , & Bryant, D. (2015). popart: Full‐feature software for haplotype network construction. Methods in Ecology and Evolution, 6(9), 1110–1116.

[ece38168-bib-0038] Librado, P. , & Rozas, J. (2009). DnaSP v5: A software for comprehensive analysis of DNA polymorphism data. Bioinformatics, 25(11), 1451–1452. 10.1093/bioinformatics/btp187 19346325

[ece38168-bib-0039] Lowe, S. , Browne, M. , Boudjelas, S. , & De Poorter, M. (2000). 100 of The World Worst Invasive Alien Species. A Selection from The Global Invasive Species Database. The Invasive Species Specialist Group, SSC, IUCN. Hollands Printing Ltd.

[ece38168-bib-0040] Macey, J. R. , Schulte, J. A. 2nd , Ananjeva, N. B. , Larson, A. , Rastegar‐Pouyani, N. , Shammakov, S. M. , & Papenfuss, T. J. (1998). Phylogenetic relationships among Agamid lizards of the Laudakia caucasia species group: Testing hypotheses of biogeographic fragmentation and an area cladogram for the Iranian Plateau. Molecular Phylogenetics and Evolution, 10(1), 118–131. 10.1006/mpev.1997.0478 9751922

[ece38168-bib-0041] Mallick, J. K. (2009). Endemic Marsh Mongoose *Herpestes palustris* (Carnivora: Herpestidae) of East Kolkata Wetlands, India: A status report. Journal of Threatened Taxa, 1(4), 215–220. 10.11609/JoTT.o1936.215-20

[ece38168-bib-0042] Marcer, A. , Méndez‐Vigo, B. , Alonso‐Blanco, C. , & Picó, F. X. (2016). Tackling intraspecific genetic structure in distribution models better reflects species geographical range. Ecology and Evolution, 6(7), 2084–2097. 10.1002/ece3.2010 27066224 PMC4768750

[ece38168-bib-0043] May, S. E. , Medley, K. A. , Johnson, S. A. , & Hoffman, E. A. (2011). Combining genetic structure and ecological niche modeling to establish units of conservation: A case study of an imperiled salamander. Biological Conservation, 144(5), 1441–1450. 10.1016/j.biocon.2011.01.013

[ece38168-bib-0044] McGarigal, K. S. , Cushman, S. , & Neel, M. (2002). FRAGSTATS: Spatial pattern analysis program for categorical maps. Computer software program produced by the authors at the University of Massachusetts, Amherst. Available at the following web site: http://www.umass.edu/landeco/research/fragstats/fragstats.html

[ece38168-bib-0045] McRae, B. H. , & Beier, P. (2007). Circuit theory predicts gene flow in plant and animal populations. Proceedings of the National Academy of Sciences, 104(50), 19885. 10.1073/pnas.0706568104 PMC2148392 18056641

[ece38168-bib-0046] Naimi, B. (2017). Pavkage Usdm: Uncertainty analysis for species distribution models. Retrieved from https://cran.r‐project.org/web/packages/usdm/usdm.pdf

[ece38168-bib-0047] Nakazato, T. , Warren, D. L. , & Moyle, L. C. (2010). Ecological and geographic modes of species divergence in wild tomatoes. American Journal of Botany, 97(4), 680–693. 10.3732/ajb.0900216 21622430

[ece38168-bib-0048] Nazarizadeh, M. , Kaboli, M. , Rezaie, H. R. , Harisini, J. I. , & Pasquet, E. (2016). Phylogenetic relationships of Eurasian Nuthatches (*Sitta europaea* Linnaeus, 1758) from the Alborz and Zagros Mountains, Iran. Zoology in the Middle East, 62(3), 217–226.

[ece38168-bib-0049] Nilson, G. , Rastegar‐Pouyani, N. , Rastegar‐Pouyani, E. , & Andrén, C. (2003). Lacertas of south and central Zagros Mountains, Iran, with description of two new taxa. Russian Journal of Herpetology, 10(1), 11–24.

[ece38168-bib-0050] Patou, M.‐L. , McLenachan, P. A. , Morley, C. G. , Couloux, A. , Jennings, A. P. , & Veron, G. (2009). Molecular phylogeny of the Herpestidae (Mammalia, Carnivora) with a special emphasis on the Asian *Herpestes* . Molecular Phylogenetics and Evolution, 53(1), 69–80. 10.1016/j.ympev.2009.05.038 19520178

[ece38168-bib-0051] Pearson, R. G. , & Dawson, T. P. (2003). Predicting the impacts of climate change on the distribution of species: Are bioclimate envelope models useful? Global Ecology and Biogeography, 12(5), 361–371. 10.1046/j.1466-822X.2003.00042.x

[ece38168-bib-0052] Peigné, S. , de Bonis, L. , Likius, A. , Mackaye, H. T. , Vignaud, P. , & Brunet, M. (2005). The earliest modern mongoose (Carnivora, Herpestidae) from Africa (late Miocene of Chad). Naturwissenschaften, 92(6), 287–292. 10.1007/s00114-005-0626-0 15864513

[ece38168-bib-0053] Peterson, A. , Soberón, J. , & Sánchez‐Cordero, V. (1999). Conservatism of ecological niches in evolutionary time. Science, 285(5431), 1265–1267. 10.1126/science.285.5431.1265 10455053

[ece38168-bib-0054] Phillips, S. J. , Anderson, R. P. , & Schapire, R. E. (2006). Maximum entropy modeling of species geographic distributions. Ecological Modelling, 190(3–4), 231–259. 10.1016/j.ecolmodel.2005.03.026

[ece38168-bib-0055] Pons, J. , Barraclough, T. G. , Gomez‐Zurita, J. , Cardoso, A. , Duran, D. P. , Hazell, S. , Kamoun, S. , Sumlin, W. D. , & Vogler, A. P. (2006). Sequence‐based species delimitation for the DNA taxonomy of undescribed Insects. Systematic Biology, 55(4), 595–609. 10.1080/10635150600852011 16967577

[ece38168-bib-0056] Posada, D. (2008). jModeltest. Version 0.1. 1. Universidad de Vigo. Available at http://darwin.uvigo.es/software/jmodeltest.html

[ece38168-bib-0057] Puillandre, N. , Lambert, A. , Brouillet, S. , & Achaz, G. (2012). ABGD, Automatic Barcode Gap Discovery for primary species delimitation. Molecular Ecology, 21(8), 1864–1877. 10.1111/j.1365-294X.2011.05239.x 21883587

[ece38168-bib-0058] Rambaut, A. , & Drummond, A. J. (2015). LogCombiner v.1.8.0. https://beast.community/logcombiner

[ece38168-bib-0059] Rambaut, A. , Drummond, A. J. , & Suchard, M. (2007). Tracer v.1. 6. https://beast.community/tracer

[ece38168-bib-0060] Raxworthy, C. J. , Ingram, C. M. , Rabibisoa, N. , & Pearson, R. G. (2007). Applications of ecological niche modeling for species delimitation: A review and empirical evaluation using day geckos (Phelsuma) from Madagascar. Systematic Biology, 56(6), 907–923. 10.1080/10635150701775111 18066927

[ece38168-bib-0061] Rezazadeh, E. , Aliabadian, M. , Darvish, J. , & Ahmadzadeh, F. (2020). Diversification and evolutionary history of brushtailed mice, Calomyscidae (Rodentia), in southwestern Asia. Organisms Diversity & Evolution, 20, 155–170. 10.1007/s13127-019-00426-y

[ece38168-bib-0062] Rice, W. R. (1989). Analyzing tables of statistical tests. Evolution, 43, 223–225. 10.1111/j.1558-5646.1989.tb04220.x 28568501

[ece38168-bib-0063] Rissler, L. J. , & Apodaca, J. J. (2007). Adding more ecology into species delimitation: Ecological niche models and phylogeography help define cryptic species in the black salamander (*Aneides flavipunctatus*). Systematic Biology, 56(6), 924–942. 10.1080/10635150701703063 18066928

[ece38168-bib-0064] Ronquist, F. , Teslenko, M. , van der Mark, P. , Ayres, D. L. , Darling, A. , Höhna, S. , Larget, B. , Liu, L. , Suchard, M. A. , & Huelsenbeck, J. P. (2012). MrBayes 3.2: Efficient Bayesian phylogenetic inference and model choice across a large model space. Systematic Biology, 61(3), 539–542. 10.1093/sysbio/sys029 22357727 PMC3329765

[ece38168-bib-0065] Ruiz‐Sanchez, E. , & Sosa, V. (2010). Delimiting species boundaries within the Neotropical bamboo Otatea (Poaceae: Bambusoideae) using molecular, morphological and ecological data. Molecular Phylogenetics and Evolution, 54(2), 344–356. 10.1016/j.ympev.2009.10.035 19897047

[ece38168-bib-0066] Salleh, F. , Ramos Madrigal, J. , Penaloza, F. , Liu, S. , Sinding, M.‐H. , Patel, R. , Martins, R. , Fickel, J. , Roos, C. , Shamsir, S. , Azman, S. , Lim, B. , Rossiter, S. , Wilting, A. , & Gilbert, M. (2017). An expanded mammal mitogenome dataset from Southeast Asia. GigaScience, 6. 10.1093/gigascience/gix053 PMC5737531 28873965

[ece38168-bib-0067] Salah, M. (2019). Seismological evidence for lithospheric low‐velocity anomalies beneath the eastern Mediterranean: Impact of tectonics. Geotectonics, 53, 617–633. 10.1134/S0016852119050054

[ece38168-bib-0068] Sborshchikov, I. M. , Savostin, L. A. , & Zonenshain, L. P. (1981). Present plate tectonics between Turkey and Tibet. Tectonophysics, 79, 45–73. 10.1016/0040-1951(81)90232-8

[ece38168-bib-0069] Scriven, J. J. , Whitehorn, P. R. , Goulson, D. , & Tinsley, M. C. (2016). Niche partitioning in a sympatric cryptic species complex. Ecology and Evolution, 6(5), 1328–1339. 10.1002/ece3.1965 26848386 PMC4730923

[ece38168-bib-0070] Shahnaseri, G. , Hemami, M.‐R. , Khosravi, R. , Malakoutikhah, S. , Omidi, M. , & Cushman, S. A. (2019). Contrasting use of habitat, landscape elements, and corridors by grey wolf and golden jackal in central Iran. Landscape Ecology, 34(6), 1263–1277. 10.1007/s10980-019-00831-w

[ece38168-bib-0071] Simberloff, D. , Dayan, T. , Jones, C. , & Ogura, G. (2000). Character displacement and release in the small Indian mongoose, *Herpestes javanicus* . Ecology, 81, 2086–2099.

[ece38168-bib-0072] Thompson, J. D. , Higgins, D. G. , & Gibson, T. J. (1994). CLUSTAL W: Improving the sensitivity of progressive multiple sequence alignment through sequence weighting, position‐specific gap penalties and weight matrix choice. Nucleic Acids Research, 22(22), 4673–4680. 10.1093/nar/22.22.4673 7984417 PMC308517

[ece38168-bib-0073] Thuiller, W. , Georges, D. , Gueguen, M. , Engler, R. , & Breiner, F. (2021). Package biomod2: Ensemble platform for species distribution modeling. Retrieved from https://cran.r‐project.org/web/packages/biomod2/biomod2.pdf

[ece38168-bib-0074] Thulin, C. G. , Simberloff, D. , Barun, A. , McCracken, G. , Pascal, M. , & Islam, M. A. (2006). Genetic divergence in the small Indian mongoose (*Herpestes auropunctatus*), a widely distributed invasive species. Molecular Ecology, 15, 3947–3956. 17054495 10.1111/j.1365-294X.2006.03084.x

[ece38168-bib-0075] Venter, O. , Sanderson, E. W. , Magrach, A. , Allan, J. R. , Beher, J. , Jones, K. R. , Possingham, H. P. , Laurance, W. F. , Wood, P. , Fekete, B. M. , Levy, M. A. , & Watson, J. E. (2018). Last of the Wild Project, Version 3 (LWP‐3): 2009 Human Footprint, 2018 Release. NASA Socioeconomic Data and Applications Center (SEDAC).

[ece38168-bib-0076] Veron, G. , & Jennings, A. P. (2017). Javan mongoose or small Indian mongoose–who is where? Mammalian Biology, 87, 62–70. 10.1016/j.mambio.2017.05.006

[ece38168-bib-0077] Wang, I. J. , & Summers, K. (2010). Genetic structure is correlated with phenotypic divergence rather than geographic isolation in the highly polymorphic strawberry poison‐dart frog. Molecular Ecology, 19(3), 447–458. 10.1111/j.1365-294X.2009.04465.x 20025652

[ece38168-bib-0078] Yusefi, G. , Faizolahi, K. , Darvish, J. , Safi, K. , & Brito, J. (2019). The species diversity, distribution, and conservation status of the terrestrial mammals of Iran. Journal of Mammalogy, 100(1), 55–71. 10.1093/jmammal/gyz002

[ece38168-bib-0079] Zhang, J. , Kapli, P. , Pavlidis, P. , & Stamatakis, A. (2013). A general species delimitation method with applications to phylogenetic placements. Bioinformatics, 29(22), 2869–2876. 10.1093/bioinformatics/btt499 23990417 PMC3810850

[ece38168-bib-0080] Ziaie, H. (2008). A field guide to the mammals of Iran. Iranian Wildlife Center.

