# Supplementary material for: Genetic structure and ecological niche segregation of Indian gray mongoose (Urva edwardsii) in Iran
Source: Ecol Evol. 2021 Sep 29;11(21):14813–27. doi: 10.1002/ece3.8168 (PMC8571580; doi:10.1002/ece3.8168)
Supplement: Supplementary file 1 — Table S1 [file ECE3-11-14813-s001.docx]

**Table S1**. Spatial autocorrelation of the predictor variables at occurrence localities of *U. edwardsii*. Values of Moran’s I range between 1 (indicating strong positive spatial autocorrelation) and 1 (indicating strong negative spatial autocorrelation) while 0 indicates a random pattern with no spatial autocorrelation (Cliff & Ord, 1981).

| **Climate variable** | **Presence localities** |
| --- | --- |
| Temperature annual range | 0.408 |
| Mean temperature of wettest quarter | 0.385 |
| Mean temperature of warmest quarter | 0.442 |
| Precipitation of wettest month | 0.357 |
| Precipitation seasonality | 0.391 |
| Precipitation of warmest quarter | 0.353 |
| Precipitation of coldest quarter | 0.306 |
| Herbaceous with sparse density of tree and shrub | 0.155 |
| Sparse herbaceous | 0.250 |
| Consolidated land | 0.212 |
| Unconsolidated land | 0.163 |
| Human footprint | 0.156 |
| Elevation | 0.443 |
| Topographic roughness | 0.166 |

Cliff, A.D. & Ord, J.K. (1981) Spatial processes: models and applications, London. Pion, Pages 266.
